# Downregulation of HNRNPK in human cancer cells inhibits lung metastasis

**DOI:** 10.1002/ame2.12090

**Published:** 2019-11-21

**Authors:** Mengyuan Li, Wenlong Zhang, Xingjiu Yang, Hongfei Liu, Lin Cao, Weisha Li, Le Wang, Guoxin Zhang, Ran Gao

**Affiliations:** ^1^ Key Laboratory of Human Disease Comparative Medicine (National Health and Family Planning Commission) The Institute of Laboratory Animal Science Chinese Academy of Medical Sciences & Peking Union Medical College Beijing P.R. China; ^2^ Beijing Engineering Research Center for Experimental Animal Models of Human Critical Diseases Beijing P.R. China

**Keywords:** HNRNPK, lung metastasis, mouse model

## Abstract

**Background:**

Lung cancer frequently occurs in the clinic, leading to poor prognosis and high mortality. Markers for early diagnosis of lung cancer are scarce, and further potential therapeutic targets are also urgently needed.

**Method:**

We established a new mouse model in which the specific gene HNRNPK (heterogeneous nuclear ribonucleoprotein K) was downregulated after administration of doxycycline. The lung metastatic nodules were investigated using bioluminescence imaging, micro‐CT, and autopsy quantification.

**Results:**

Compared with the short hairpin negative control group, less lung metastatic nodules were formed in the short hairpin RNA group.

**Conclusion:**

Downregulation of HNRNPK in cancer cells can inhibit lung metastasis.

## 1 INTRODUCTION

Lung cancer is the leading cause of cancer‐related death worldwide.^1^ Non‐small cell lung cancer is the most common type of lung cancer, accounting for 80%‐85% of the total number of lung cancers. Early detection can significantly improve the survival rate; however, approximately 75% of patients are diagnosed in later stages, which hinders curative therapy. The survival time of lung cancer patients is still very low,^2^ with the 5‐year survival rate being only 15%, and early detection remains challenging. Therefore, it is important to identify new prognostic markers and develop novel treatment strategies for lung cancer.

HNRNPK is a highly conserved RNA‐ and DNA‐binding protein^3^ located on chromosome 9 q21.32‐q21.33 containing three consecutive K homology (KH) domains, a nuclear localization signal (NLS), and a nuclear shuttling (KNS) domain. KH domains are responsible for the binding of RNA or single‐stranded DNA consisting of 65‐70 amino acids.^4^ The NLS domain regulates the protein's transportation from the cytoplasm to the nucleus, while the KNS domain promotes bidirectional translocation via the nuclear pore complex.^5^

It has been shown that dysregulation of HNRNPK is associated with tumor development, progression, and prognosis. For instance, HNRNPK can promote c‐myc transcription by binding to poly (C) of the c‐myc promoter both in vitro and in vivo,^6^, ^7^ and high expression of HNRNPK is usually accompanied by high levels of c‐myc in breast cancer, prostate cancer, and melanoma tissues.^8^, ^9^, ^10^ In contrast, HNRNPK may also act as a tumor suppressor, as haploinsufficient mice more easily form malignant tumors.^11^, ^12^, ^13^

Moreover, HNRNPK also plays an important role in cell homeostasis. As a coactivator of p53, HNRNPK regulates DNA damage repair. DNA damage enables HNRNPK to be recruited to the promoter of the p53 downstream gene, thereby promoting the expression of p21, HDM2, C/EBPα, and C/EBPβ. Downregulation of HNRNPK reduces p53 transcription, leading to DNA damage‐induced cell cycle arrest.^11^ In addition, HNRNPK can bind to the promoter region and to the 3′ untranslated region of p21 messenger ribonucleic acid (mRNA), which inhibits translation of p21 and increases translation initiation, cell division, and tumor formation.^14^, ^15^

Although previous research has revealed some effects of HNRNPK in cancer and normal cells, the exact role of HNRNPK in lung cancers is still unknown and requires further investigation. We established a new human tumor cell line with reduced expression of HNRNPK. In vivo data showed that downregulation of HNRNPK significantly reduced the formation of metastatic lung tumor nodules. Notably, downregulation of HNRNPK was performed 1 week after tumor implantation by induction with doxycycline (DOX). This research paves the way for clinical applications targeting HNRNPK and improved therapies.

## 2 MATERIALS AND METHODS

### 2.1 Cell culture and transfection

Downregulated HNRNPK‐related lentivirus (LV‐HNRNPK‐RNAi) and negative control (LV‐NC‐RNAi) vectors were procured from GeneChem Co. Ltd. The A549 cell line was purchased from the Institute of Basic Medical Sciences, Chinese Academy of Medical Sciences. Cells at 70%‐80% confluence were transfected with lentivirus in six‐well plates in accordance with the manufacturer's recommendations. After 72 hours, cells were continuously cultured with 6 μg/mL puromycin (P8833; Sigma) for 48 hours to select the clones with stably downregulated HNRNPK. Then, A549‐luc‐H1TetO‐shRNA‐HNRNPK and A549‐luc‐H1TetO‐shRNA‐NC cell lines were cultured in RPMI 1640 (11875‐093; Gibco) with 10% fetal bovine serum (10099‐141C; Gibco), 1% penicillin‐streptomycin (15140‐122; Gibco), and 3 μg/mL puromycin in a cell culture incubator with 5% CO_2_ at 37°C. To specifically downregulate the expression of HNRNPK, 5 μg/mL DOX (REVG1004; GeneChem Co. Ltd.) was used.

### 2.2 Animals

Animals had free access to food and water and were maintained under controlled temperature, humidity, and light conditions. All procedures were approved by the Biomedical Ethical Committee of the Chinese Academy of Medical Sciences (GR17001). Six‐ to eight‐week‐old female BALB/C‐nu mice were purchased from Beijing Huafukang Biotechnology. Six mice were used for each group. 1 × 10^6^ A549‐luc‐H1TetO‐shRNA‐HNRNPK or A549‐luc‐H1TetO‐shRNA‐NC (negative control) cells were injected through the mouse tail vein. One week later, the mice were fed 200 ppm DOX (C11300‐200i; Research Diets). In addition, mouse body weight was recorded weekly. Lung tumor masses were detected by bioluminescence imaging every week. Six weeks later, all mice were killed, and the lungs were harvested to quantify the number of metastatic nodules.

### 2.3 Reverse trancription polymerase chain reaction

Total RNA was extracted from cultured A549 cells in accordance with the instructions of the Trizol reagent kit (15996‐018; Invitrogen). Complementary DNA (cDNA) was synthesized using a RevertAid First Strand cDNA Synthesis Kit (K1622; Thermo). Quantitative PCR was performed using the CFX Connect^TM^ Real‐Time PCR system (Bio‐Rad) and SYBR Green Real‐time PCR Master Mix (QPK‐201; TOYOBO). HNRNPK and glyceraldehyde‐3‐phosphate dehydrogenase (GAPDH) primers used for quantitative real‐time PCR were synthesized by Invitrogen and are listed in Table 1. To avoid bias, each assay was performed in triplicate, and the relative gene expression was calculated by the comparative cycle threshold ($2^{-\Delta \Delta C_{t}}$) method following the manufacturer's instructions.

**Table 1:** Primer sequences for the real‐time PCR assay

Names	Sequence (5′‐3′)
HNRNPK‐Forward	AGGTCGGTGTGAACGGATTTG
HNRNPK‐Reverse	TGTAGACCATGTAGTTGAGGTCA
GAPDH‐Forward	AGACCTGGAGACCGTTAC
GAPDH‐Reverse	ATAAGCCATCTGCCATTC

### 2.4 Western blot assay

First, cells were homogenized in radio‐immunoprecipitation assay lysis buffer (R0010; Solarbio) containing 1% phenylmethanesulfonyl fluoride on ice for 30 minutes. Total protein concentrations were estimated using the Pierce bicinchoninic acid protein assay kit (23225; Thermo). Then, equal amounts of cell lysates were loaded and separated by 12% sodium dodecyl sulfate polyacrylamide gel electrophoresis. Then, proteins were transferred to nitrocellulose membranes for 1 hour with a 300 mA current. Next, the membranes were incubated with a 1:1000 dilution of anti‐HNRNPK antibody (ab52600; Abcam) and a 1:10 000 dilution of anti‐β‐actin antibody (ab49900; Abcam) overnight at 4°C with 5% nonfat milk, washed three times with tris‐buffered saline tween 20 buffer, and then incubated with a 1:10 000 dilution of horseradish peroxidase‐conjugated goat anti‐rabbit Immunoglobulin G (ZDR‐5307; ZSGB‐Bio) for 1 hour at room temperature. The membranes were visualized with an emitter coupled logic detection system (Thermo).

### 2.5 Bioluminescence imaging

Biolayer interferometry was performed once a week until the end of the study using an in vivo imaging system‐Lumina II (Perkin Elmer), generating a pseudocolored image representing light intensity and superimposed over a grayscale reference image. Each mouse was intraperitoneally injected with 150 mg/kg luciferin potassium salt (115144‐35‐9; Fanbo). Ten minutes later, the mice were anesthetized using 1.5% isoflurane. To maintain body temperature, mice were placed on a thermostatically controlled heating pad (37°C) during imaging. Acquisition binning and duration were set according to tumor activity. Signal intensity was quantified as the total flux (photons/s) within regions of interest drawn manually around the tumor area using Living Image 4.0 software (Perkin Elmer). Signals from both prone and supine positions were obtained.

### 2.6 Micro‐CT

Micro‐CT scanning was performed using a FLE computed tomography system (InSyTe^TM^; TriFoil Imaging) with 45 kVp X‐ray voltage, 1100 ms exposure time, and a pitch of 1. The total projection of 240 slices over 360° of rotation was acquired. Projection data were re‐binned in a ratio of 1:4 and reconstructed with a Butterworth filter.

### 2.7 Statistical analysis

GraphPad Prism 5.0 was used for statistical analysis. All the data were expressed as the mean ± SEMs. Statistical differences between two groups were determined by Student's *t* test, while one‐way ANOVA was used for multiple groups. *P* < .05 was considered statistically significant.

## 3 RESULTS

### 3.1 Establishment of lentiviral vector for the short hairpin RNA (sh‐HNRNPK) sequence

**Figure 1:**
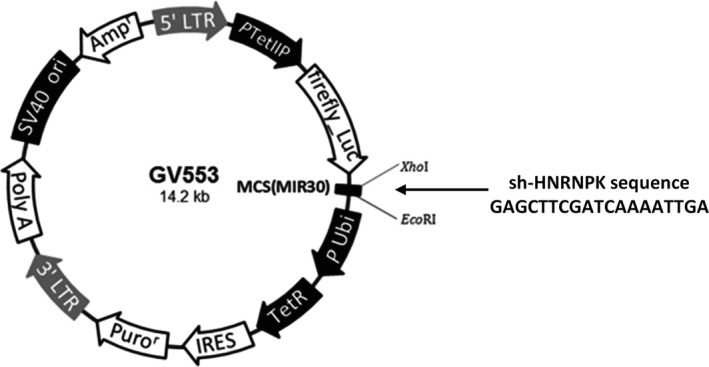
Construction of a lentiviral vector for the sh‐HNRNPK sequence. The sh‐HNRNPK sequence was cloned into the multiple cloning site of the GV553 lentiviral vector

A PCR product with artificial *Xho*I and *Eco*RI enzyme restriction sites at the 5′ and 3′ ends containing the full‐length coding sequence of HNRNPK‐RNAi (69976‐1) was amplified and subsequently cloned into the multiple cloning site of the lentiviral expression vector GV553. The resulting construct was verified by restriction enzyme digest and direct sequencing (Figure 1).

### 3.2 HNRNPK is downregulated in a DOX‐dependent manner

**Figure 2:**
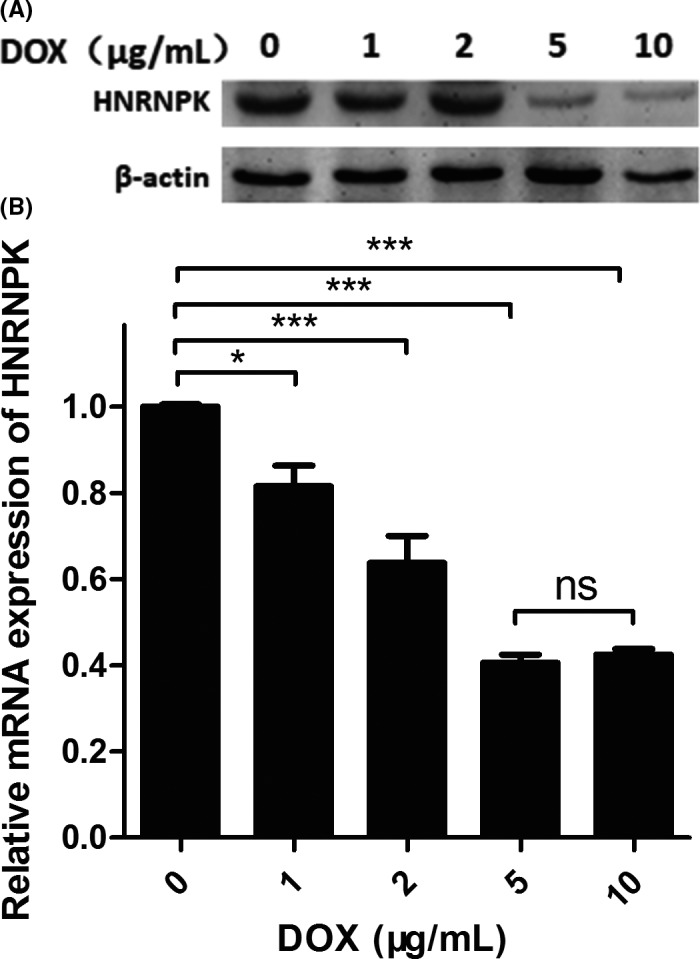
Doxycycline (DOX) decreased HNRNPK expression in A549 cells. A, Western blot results indicated that HNRNPK expression was downregulated after DOX administration in a dose‐dependent manner. B, HNRNPK mRNA was significantly decreased after DOX induction, and a higher dose of DOX did not lead to further HNRNPK downregulation

To investigate whether HNRNPK expression was regulated by the siRNA, an in vitro assay was performed in lentiviral vector‐transfected A549 lung cancer cells. As Figure 2 shows, HNRNPK was downregulated at both the mRNA and protein levels after DOX induction. As shown in Figure 2A, Western blot results indicated that HNRNPK was downregulated in a DOX‐dependent manner. HNRNPK expression dramatically decreased after 5 and 10 μg/mL DOX treatments compared with the levels seen in the negative control and with lower DOX concentrations. This result was confirmed by RT‐PCR, as shown in Figure 2B: 5 and 10 μg/mL DOX significantly reduced the mRNA expression of HNRNPK. Although 1 and 2 μg/mL DOX also decreased HNRNPK expression, the extent of reduction was not as high as was seen with 5 μg/mL. Surprisingly, 10 μg/mL DOX did not further reduce HNRNPK levels.

### 3.3 Downregulation of HNRNPK decreased lung metastasis

**Figure 3:**
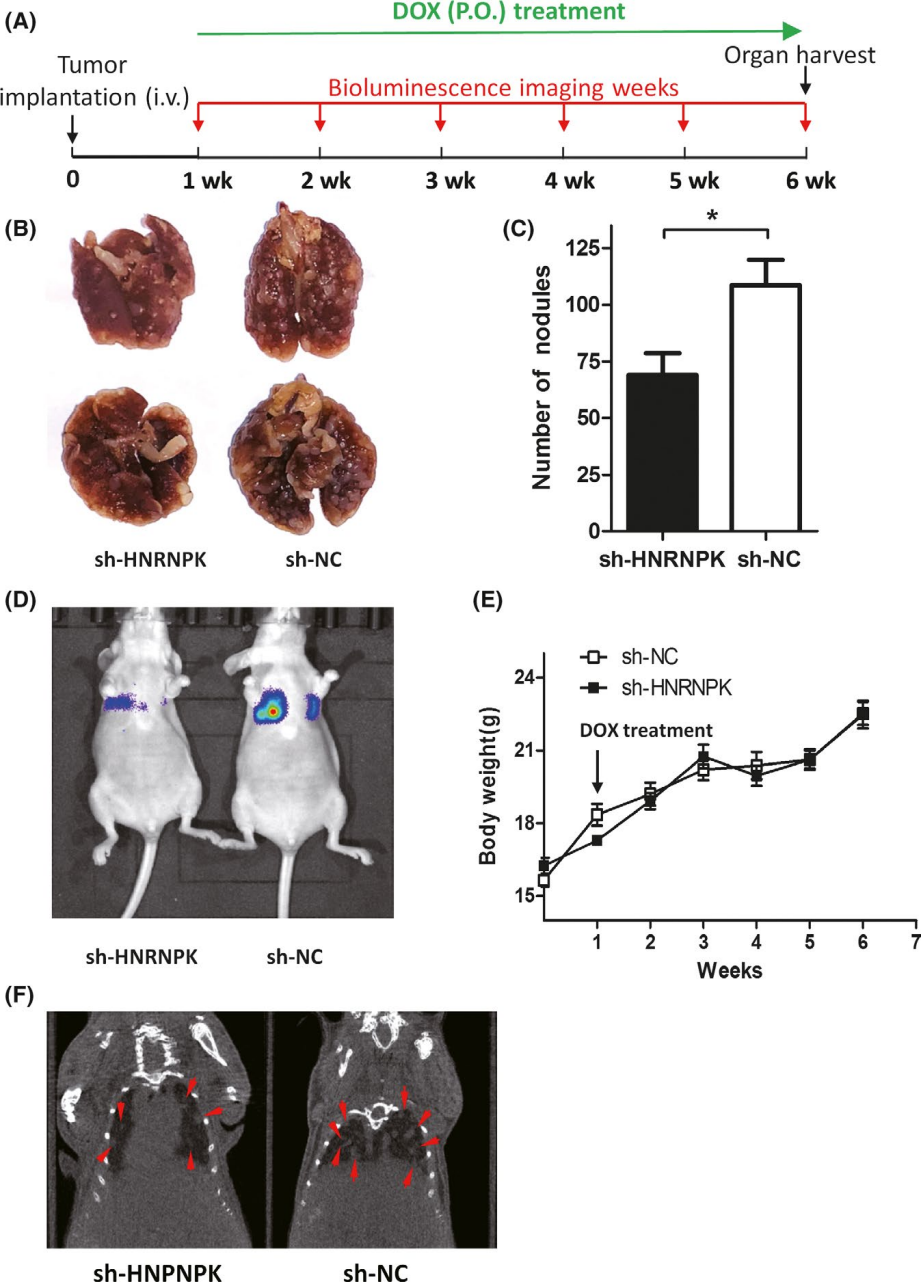
Downregulation of HNRNPK inhibits lung metastasis. A, Time course of the mouse experiment. B and C, Metastatic nodules were significantly decreased in sh‐HNRNPK group. D, Bioluminescence imaging comparing single sh‐HNRNPK and short hairpin negative control (sh‐NC) cancer cells in lung metastases. E, Mouse body weight was similar in the sh‐HNRNPK and sh‐NC groups. F, Micro‐CT images showing the metastatic tumor mass in the lung in both groups

To reveal the role of HNRNPK, a mouse lung metastasis model was used (Figure 3). Figure 3A illustrates the time course of the experiment. A549 tumor cells were intravenously injected through the tail vein, and then 1 week later, DOX treatment was started to induce the downregulation of HNRNPK expression. Simultaneously, metastatic foci in the lung were followed by bioluminescence imaging and micro‐CT in vivo. Figure 3D,F illustrates that a lower signal was obtained in the sh‐HNRNPK group than in the control group 6 weeks after tumor implantation. At the end of the experiment, mice were killed, and the lungs were harvested (Figure 3A). Additionally, the metastatic foci were calculated (Figure 3B), and the quantification results indicated that in the sh‐HNRNPK group, the number of metastatic nodules was significantly decreased. However, this did not lead to bodyweight changes (Figure 3E).

### 3.4 Mouse model schematic

**Figure 4:**
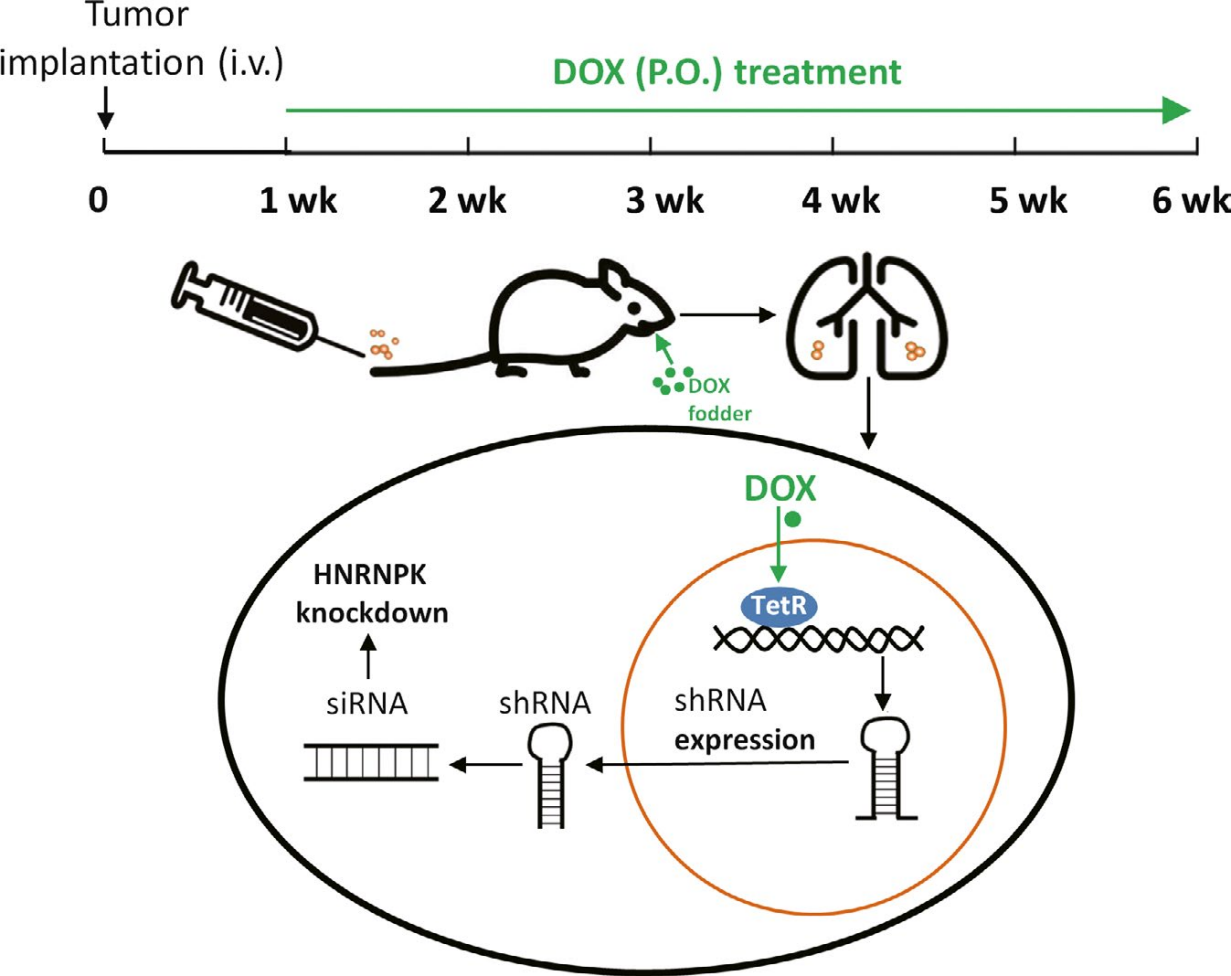
Schematic of the newly established mouse model. A time course and the mechanism of the mouse model are shown

Figure 4 shows a schematic of the mouse model. One week after intravenous injection of tumor cells, DOX treatment was started to induce the activation of TetR, and then expression of the shRNA targeting HNRNPK was activated. Thus, the HNRNPK gene was specifically knocked down in the mouse lung.

## 4 DISCUSSION

Clinical data show that HNRNPK is overexpressed in lung cancer tissues,^16^ and high expression of HNRNPK is usually associated with poor prognosis.^15^, ^17^ This suggests that HNRNPK may play an important role in the progression of lung cancer cells.

Many scientists have focused on HNRNPK and attempted to reveal the role of HNRNPK in cancer progression. Gallardo et al generated a mouse model harboring an HNRNPK‐knockout allele (Hnrnpk^+/−^) and demonstrated that HNRNPK is a tumor suppressor gene involved in hematologic malignancies.^13^ A study from Huang et al indicated that HNRNPK overexpression inhibits gastric cancer by regulating the p53 signaling pathway by establishing a subcutaneous tumorigenesis model in nude mice.^18^ Moreover, Li et al subcutaneously injected neuroblastoma cells that were stably transfected with pancEts‐1‐specific shRNAs into nude mice.^19^ Their data indicated that HNRNPK was highly expressed and predicted poor clinical outcome in neuroblastoma patients.

Although these experiments advanced the understanding of the function of HNRNPK, they failed to establish a mouse model to unearth its role in lung metastasis.

We established a new tumor cell line in which HNRNPK can be downregulated in a DOX‐dependent manner. Notably, HNRNPK can be downregulated at a specific time point. The biggest advantage of our newly established model is that HNRNPK is downregulated 1 week after tumor implantation, which, to a large extent, stimulates the clinical treatment process.

Our results showed that downregulation of HNRNPK significantly reduced lung metastasis formation, laying the foundation for novel inhibitor development. Blockade of HNRNPK function using inhibitors may prevent lung cancer formation. Additionally, the expression of HNRNPK in lung cancer cells may help to determine the prognosis of patients.

Although our model reveals some mechanisms surrounding the role of HNRNPK in lung cancer metastasis, the mechanism and factors involved in the regulation of HNRNPK expression are still unknown and need to be investigated in the future.

Overall, we established a new model in which HNRNPK expression in cancer cells was specifically downregulated and downregulation of HNRNPK significantly inhibited lung metastasis. This indicates that HNRNPK may serve as a target for the development of new therapeutic strategies for the treatment of lung metastasis.

## CONFLICT OF INTEREST

None.

## AUTHOR CONTRIBUTIONS

RG and ML designed the experiment. XY, HL, LC, WL, LW, and GZ performed the experiment. ML and WZ carried out the data analysis. RG, LM, and WZ prepared the manuscript. All authors read and approved the final manuscript.
